# Genome sequencing and analysis of the paclitaxel-producing endophytic fungus *Penicillium aurantiogriseum* NRRL 62431

**DOI:** 10.1186/1471-2164-15-69

**Published:** 2014-01-25

**Authors:** Yanfang Yang, Hainan Zhao, Roberto A Barrero, Baohong Zhang, Guiling Sun, Iain W Wilson, Fuliang Xie, Kevin D Walker, Joshua W Parks, Robert Bruce, Guangwu Guo, Li Chen, Yong Zhang, Xin Huang, Qi Tang, Hongwei Liu, Matthew I Bellgard, Deyou Qiu, Jinsheng Lai, Angela Hoffman

**Affiliations:** 1 State Key Laboratory of Tree Genetics and Breeding, The Research Institute of Forestry, Chinese Academy of Forestry, Beijing 100091, China; 2 College of Agricultural Biotechnology, China Agriculture University, Beijing 100094, China; 3 Centre for Comparative Genomics, Murdoch University, Perth, WA 6150, Australia; 4 Department of Biology, East Carolina University, Greenville, NC 27858, USA; 5 School of Life Sciences, Southwest Forestry University, Kunming 650224, China; 6 Kunming Institute of Botany, Chinese Academy of Sciences, Kunming 650201, China; 7 CSIRO Plant Industry, Canberra ACT 2001, Australia; 8 Department of Chemistry and Department of Biochemistry and Molecular Biology, Michigan State University, East Lansing, MI 48824, USA; 9 Department of Chemistry, University of Portland, Portland, OR 97203, USA; 10 BGI-Shenzhen, Shenzhen 518000, China; 11 Department of Plant Pathology, China Agricultural University, Beijing 100094, China; 12 Institute of Plant Quarantine, Chinese Academy of Inspection and Quarantine, Beijing 100029, China; 13 Hunan Provincial Key Laboratory of Crop Germplasm Innovation and Utilization and National Chinese Medicinal Herbs (hunan) Technology Center, Hunan Agricultural University, Changsha 410128, China

**Keywords:** *Penicillium aurantiogriseum* NRRL 62431, Paclitaxel, Taxol™, Endophytic fungi, Genome sequence, Horizontal gene transfer

## Abstract

**Background:**

Paclitaxel (Taxol™) is an important anticancer drug with a unique mode of action. The biosynthesis of paclitaxel had been considered restricted to the *Taxus* species until it was discovered in *Taxomyces andreanae*, an endophytic fungus of *T. brevifolia*. Subsequently, paclitaxel was found in hazel (*Corylus avellana* L.) and in several other endophytic fungi. The distribution of paclitaxel in plants and endophytic fungi and the reported sequence homology of key genes in paclitaxel biosynthesis between plant and fungi species raises the question about whether the origin of this pathway in these two physically associated groups could have been facilitated by horizontal gene transfer.

**Results:**

The ability of the endophytic fungus of hazel *Penicillium aurantiogriseum* NRRL 62431 to independently synthesize paclitaxel was established by liquid chromatography-mass spectrometry and proton nuclear magnetic resonance. The genome of *Penicillium aurantiogriseum* NRRL 62431 was sequenced and gene candidates that may be involved in paclitaxel biosynthesis were identified by comparison with the 13 known paclitaxel biosynthetic genes in *Taxus*. We found that paclitaxel biosynthetic gene candidates in *P. aurantiogriseum* NRRL 62431 have evolved independently and that horizontal gene transfer between this endophytic fungus and its plant host is unlikely.

**Conclusions:**

Our findings shed new light on how paclitaxel-producing endophytic fungi synthesize paclitaxel, and will facilitate metabolic engineering for the industrial production of paclitaxel from fungi.

## Background

Paclitaxel is an important anticancer diterpenoid discovered in the bark of the yew *Taxus brevifolia*[1] and its chemical structure was elucidated in 1971 [2]. It can inhibit the division of actively growing tumor cells by preventing microtubule depolymerization [3] and has become increasingly important in the treatment of a number of major cancers. Unfortunately, yew trees grow slowly and large amounts of bark are required for paclitaxel production [4]. Various attempts to obtain alternative sources of paclitaxel have been made with some success [5-9], and many pharmaceutical companies now employ semisynthetic techniques using the taxane skeleton obtained from plants. Biosynthesis of paclitaxel in *Taxus* is thought to involve 19 steps from geranylgeranyl diphosphate (in Additional file 1: Figure S1), and 13 paclitaxel biosynthetic genes have been identified (in Additional file 2: Table S1) [10]. Since the discovery of the paclitaxel-producing endophytic fungus *Taxomyces andreanae* from *T. brevifolia*[11], more than 20 genera of paclitaxel-producing fungi have been isolated from *Taxus* and non-*Taxus* plant species [12-14]. Low productivity of paclitaxel in endophytic fungi prevents these organisms from being used in commercial production of paclitaxel, and has raised the unlikely hypothesis that these fungi do not synthesize paclitaxel independently, but instead accumulate it in their cell wall from *Taxus* cells [15]. This highlights the need to study the genes that govern paclitaxel biosynthesis in endophytic fungi and their evolutionary origin [16]. PCR-based screening using the *Taxus* nucleotide sequence for taxadiene synthase (TS), a unique gene in the formation of the taxane skeleton, has been used to screen for endophytic fungi with the potential to synthesize paclitaxel, and has indicated that the gene sequences are highly conserved between plant and endophytic fungi [12]. However, a recent PCR based study using primers for TS and 10-deacetylbaccatin III-10-O-acetyltransferase (DBAT) on 11 fungal isolates from *T. media* with diverse genotypes, did not find high homology between plant and fungal genes [17]. Also Heinig et al. [15] isolated several endophytic fungi from *Taxus* spp. including EF0021 (tentatively identified as *Phialocephala fortinii*) that could not independently synthesize paclitaxel, and did not possess genes with significant similarity to known paclitaxel biosynthetic genes. Fungal isolates from the *Fusarium solani* species complex have been reported to synthesize paclitaxel [18], and a genome sequence has been constructed for a member of this complex [19]. However, the ability of this *F. solani* isolate to synthesize paclitaxel is unknown. To date, neither global identification nor evolutionary analyses have been performed on endophytic fungi demonstrated to independently synthesize paclitaxel. Insights into the genes and origin of the complete pathway could provide information on the origin of endophytic fungal genes in the paclitaxel biosynthetic pathway. This information could also facilitate metabolic engineering for the industrial production of paclitaxel from fungi.

Here, we report the genome sequence of *Penicillium aurantiogriseum* NRRL 62431, an endophytic fungus of hazel that we have confirmed to independently synthesize paclitaxel, and we have identified a large set of potential genes involved in paclitaxel biosynthesis. These candidate paclitaxel biosynthetic genes are significantly different from those found in the *Taxus* genus and seem to have evolved independently, indicating that horizontal gene transfer is an unlikely explanation. This genomic information helps elucidate the molecular mechanisms underlying the synthesis of paclitaxel in endophytic fungi and will make it possible to realize the full potential of *P. aurantiogriseum* NRRL 62431 as a source of industrial paclitaxel.

## Results

### Genome sequence assembly and annotation

**Figure 1:**
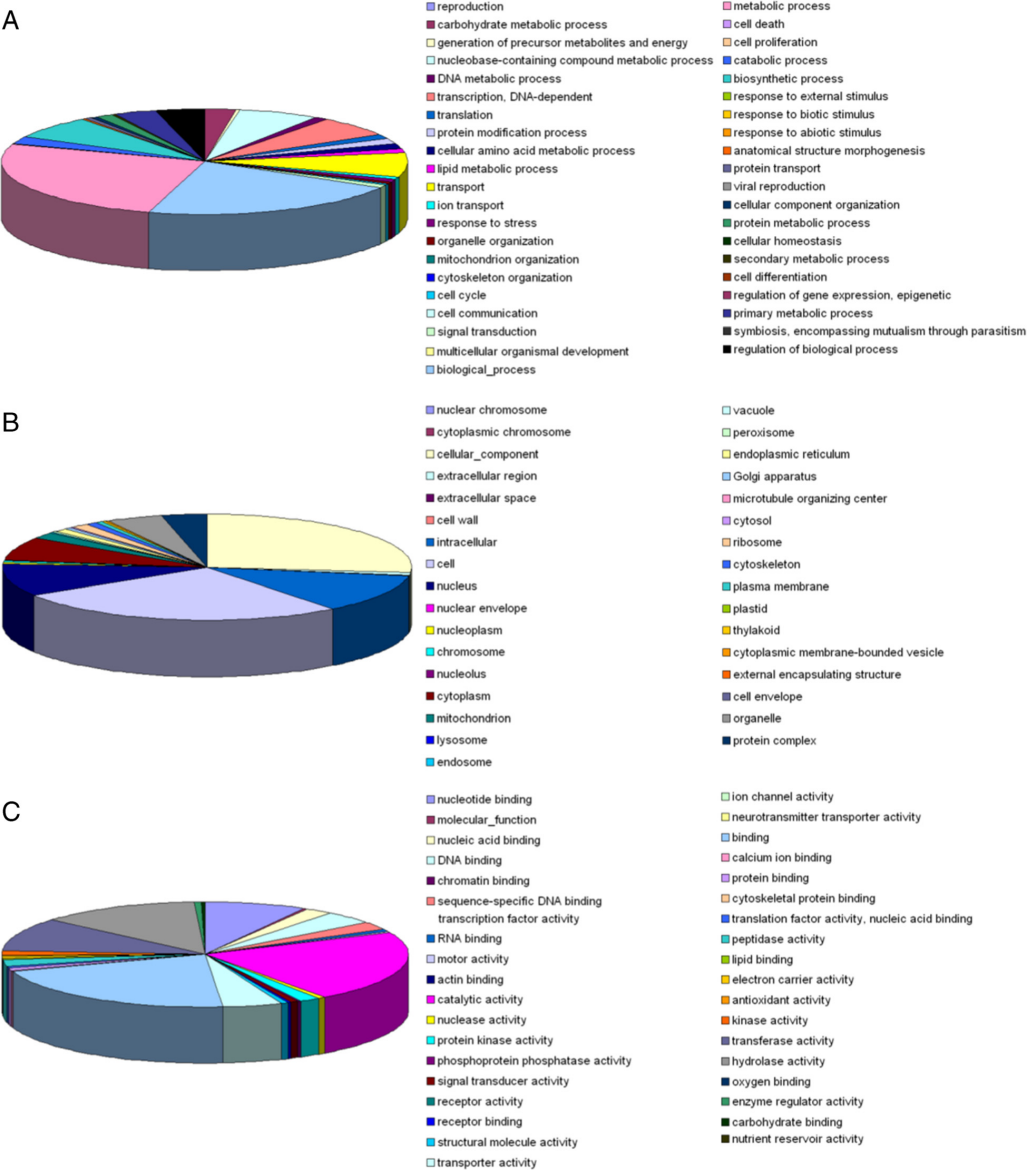
**Gene ontology classification of *P. aurantiogriseum*. (A)** Biological Process. **(B)** Cellular Component. **(C)** Molecular Function.

We isolated an endophytic *P. aurantiogriseum* fungus, NRRL 62431, from hazel and demonstrated that it can produce paclitaxel by comparing our LC-MS and ^1^H NMR data with the reported the LC-MS and ^1^H NMR data of paclitaxel [20] (Table 1, in Additional file 1: Figure S2). To investigate the paclitaxel biosynthetic genes and their evolutionary origin, we sequenced the genome of *P. aurantiogriseum* NRRL 62431. A total of 59,951,610 100-nt paired-end reads were obtained and assembled into 44,061 contigs that yielded a genome size of 32.7 Mb (Table 2). We used GeneMark [21], TWINSCAN [22] and GeneWise [23] to predict genes in *P. aurantiogriseum* NRRL 62431. The final gene set contains 11,476 genes. Gene ontology analysis categorized the gene set into 110 functional groups (Figure 1, Additional file 2: Table S2). Subsets of these functional groups were annotated as part of the ‘metabolic process’ (6,296 genes) or ‘secondary metabolic process’ (8 genes) categories. KEGG analysis assigned 11,476 genes to 284 pathways. Among them, 14 genes were found to be involved in the biosynthesis of terpenoid backbone, 17 genes in phenylalanine, tyrosine and tryptophan biosynthesis and 17 genes in phenylalanine metabolism. Transcription factor analysis revealed that 462 transcription factors were found in the genome of *P. aurantiogriseum* NRRL 62431 including C2H2, C6, Zn(II)2Cys6, GATA , HACA, APSES, HLH, bZIP, STP8, NF-Y, SRE, CP2, PHD, RFX (in Additional file 3: Data S1). Analysis of membrane transporters in the genome of *P. aurantiogriseum* NRRL 62431 identified a total of 113 predicted multidrug transporters that are presumably involved in transportation and detoxification of secondary metabolites (in Additional file 3: Data S2). Among them, 93 belong to ABC transporters (ABC multidrug transporters).

**Table 1:** ^**1**^**H nmr evidence for the presence of paclitaxel extracted from *P. aurantiogriseum *** **NRRL 62431 culture medium**


**Carbon**	**Literature values **^**20**^	**Fungal extract**
C-2	5.62 d *J* = 7	5.683 d *J* = 7
C-3	3.80 d *J* = 7	3.810 d *J* = 7
C-5	4.92 dd *J* = 2,8	4.957 dd *J* = 2,7
C-6	2.50 m	2.551 m
	1.82 m	1.820 m
C-7	4.33 m	4.317 m
C-10	6.26 s	6.272 s
C-13	6.13 t	6.215 t *J* = 8
C-14	2.5 m	2.513 m
C-16	1.25 s	1.245 s
C-17	1.14 s	1.146 s
C-18	1.78 s	1.795 s
C-19	1.67 s	1.687 s
C-20	4.17 d *J* = 8	4.188 d *J* = 8
	4.27 d *J* = 8	4.296 d *J* = 8
C-20 Bz	7.4 m	7.441 m
	8.11 dd	8.146 dd *J* = 2,8
OAC	2.23 s	2.241 s
	2.38 s	2.390 s
C-2′	4.71 d *J* = 3	4.791 d *J* = 3
C-3′	5.72 dd *J* = 3,9	5.800 dd *J* = 3,9
C-3′ Ph	7.4 m	7.425 m
C-3′ NH	7.00 d *J* = 9	6.969 d *J* = 9
C-3′Bz	7.4 m	7.501 m
	7.7 dd	7.753 dd

Chemical shifts (*δ*) expressed in ppm relative to TMS with coupling constants (*J*) in Hz.

**Table 2:** **Summary of *Penicillium aurantiogriseum *** **NRRL 62431 genome assembly**

**Number of 100 nt pair-end reads**	**59,951,610**
Total raw bases	5,995,161,000
N50	19 kb
Maximum length of contigs	109,027 bp
Total length of assembly	32.7 Mb
Number of contigs	44061
Number of contigs >1 Kb	2,701
Nucleotides of contigs >1 Kb	30.8 Mb
Number of contigs >10 Kb	1110
Nucleotides of contigs >10 Kb	23.7 Mb

Paired end reads derived from a 280 bp-insert library were sequenced using Illumina/GAii technology.

In order to identify genes involved in paclitaxel biosynthesis in *P. aurantiogriseum*, a protein search (BLASTP) was performed against the genome of *P. aurantiogriseum* NRRL 62431 using the 13 reported paclitaxel biosynthetic genes in *Taxus*. This search revealed putative homologs to 7 genes encoding phenylalanine aminomutase (PAM), geranylgeranyl diphosphate synthase (GGPPS), taxane 5α-hydroxylase (T5OH), taxane 13α-hydroxylase (T13OH), taxane 7β-hydroxylase (T7OH), taxane 2α-hydroxylase (T2OH) and taxane 10β-hydroxylase (T10OH) of *Taxus* (in Additional file 3: Data S3). In addition, an acyltransferase (PAU_P11263) was identified in the *P. aurantiogriseum* NRRL 62431 gene set by BLASTp search against GenBank databases.

### Comparative analysis of paclitaxel biosynthetic genes between *P. aurantiogriseum* NRRL 62431 and its host

Potential paclitaxel biosynthetic gene homologs with identity > 30% to the 13 reported paclitaxel biosynthetic genes were found in the paclitaxel-producing hazel [24,25]. The most conserved genes were *GGPS* and *PAM* with amino acid identities of 62% and 63%, respectively (in Additional file 3: Data S4). Comparison of the paclitaxel biosynthetic gene candidates in host hazel (in Additional file 3: Data S5) against *P. aurantiogriseum* NRRL 62431 genome showed that their paclitaxel biosynthetic genes were not highly conserved, sharing only 21% to 62% sequence identities (in Additional file 3: Data S6). Another strain of endophytic fungus *P. aurantiogriseum* was also isolated from the host plant *Taxus baccata* and was shown to synthesize taxane (10-deacetylbaccatin III) [26]. We compared *P. aurantiogriseum* NRRL 62431 genome against the paclitaxel genes in *T. baccata* (in Additional file 3: Data S7) and again found paclitaxel biosynthetic gene candidates in *P. aurantiogriseum* NRRL 62431 and paclitaxel biosynthetic genes *in T. baccata* were quite different, only 19% to 65% identical in amino acid sequences (in Additional file 3: Data S8).

### Comparative analysis of *P. aurantiogriseum* NRRL 62431 with an endophytic fungus EF0021 (*Phialocephala fortinii)*

Recently the genome of an endophytic fungus EF0021 isolated from *Taxus spp*. that was incapable of independent paclitaxel synthesis was sequenced [15]. Comparison of the paclitaxel biosynthetic candidate genes from *P. aurantiogriseum* NRRL 62431 with EF0021 revealed only potential similarity to *PAM* (43% identity over 622 nucleotides), *GGPPS* (62% highest identity over 451 nucleotides), and p450 (48% highest identity over 534 nucleotides) (in Additional file 3: Data S9).

### Phylogenetic analysis of *P. aurantiogriseum* NRRL 62431

**Figure 2:**
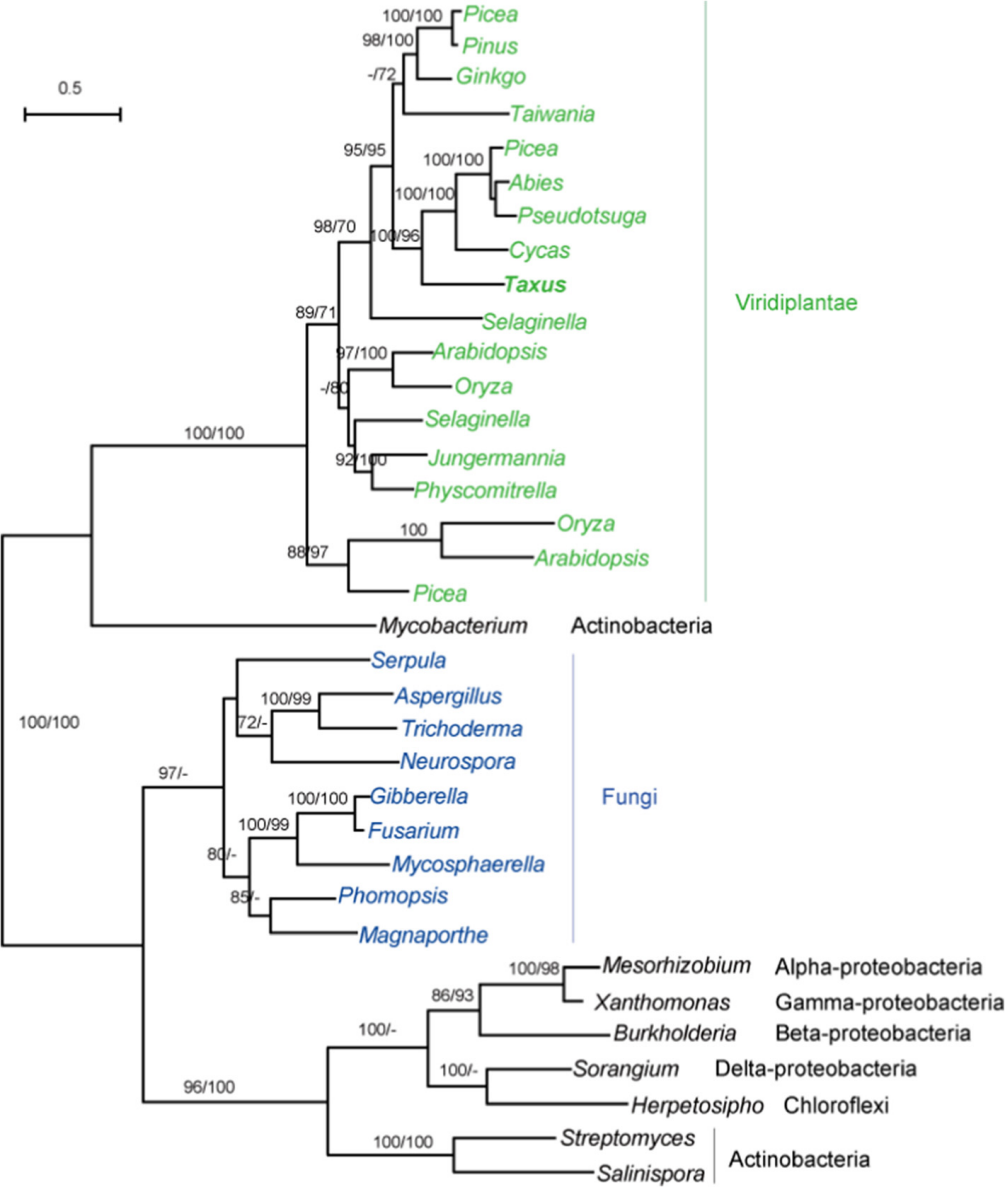
**Molecular phylogeny of the N terminal cyclase domain in TS proteins.** Numbers above branches indicate bootstrap values from maximum likelihood and distance analyses, respectively. Dashes indicate bootstrap values lower than 70%. The taxa belonging to Viridiplantae and Fungi are shown in green and blue, respectively.

Comparison of the *P. aurantiogriseum* NRRL 62431 and *F. solani* genome sequences did not reveal any significant similarity to taxadiene synthase (*TS*) in *Taxus* by BLASTp search. Position-Specific Iterative BLAST (PSI-BLAST) uses a list of all known closely related proteins to find more distant relatives and searching against GenBank database revealed homologs in some fungi and prokaryotes to the N terminal cyclase domain of *TS* in *Taxus*. Interestingly, one gene from the bacterial genus *Mycobacterium* showed high similarity to the plant *TS*, and their close relationship was further supported by the phylogenetic analysis (Figure 2), which implies the potential lateral gene transfer from plants to mycobacteria. The phylogenetic analysis also clearly showed that land plants, fungi, mycobacterium, and other bacteria formed three separate clades, which suggest that no recent gene transfer from the plant hosts to endophytic fungi has taken place. Wildung et al. found TS includes an N-terminal targeting sequence for localization and processing in the plastids [27]. This makes the gene transfer from endophytic fungi to plant less likely (Figure 2). The absence of a homolog in the paclitaxel-producing endophytic fungi *P. aurantiogriseum* NRRL 62431 and *F. solani* to *TS* in *Taxus* suggests that *P. aurantiogriseum* NRRL 62431 and *F. solani* may have a unique enzyme catalyzing the reaction towards taxadiene. This phenomenon is important and deserves further investigation.

**Figure 3:**
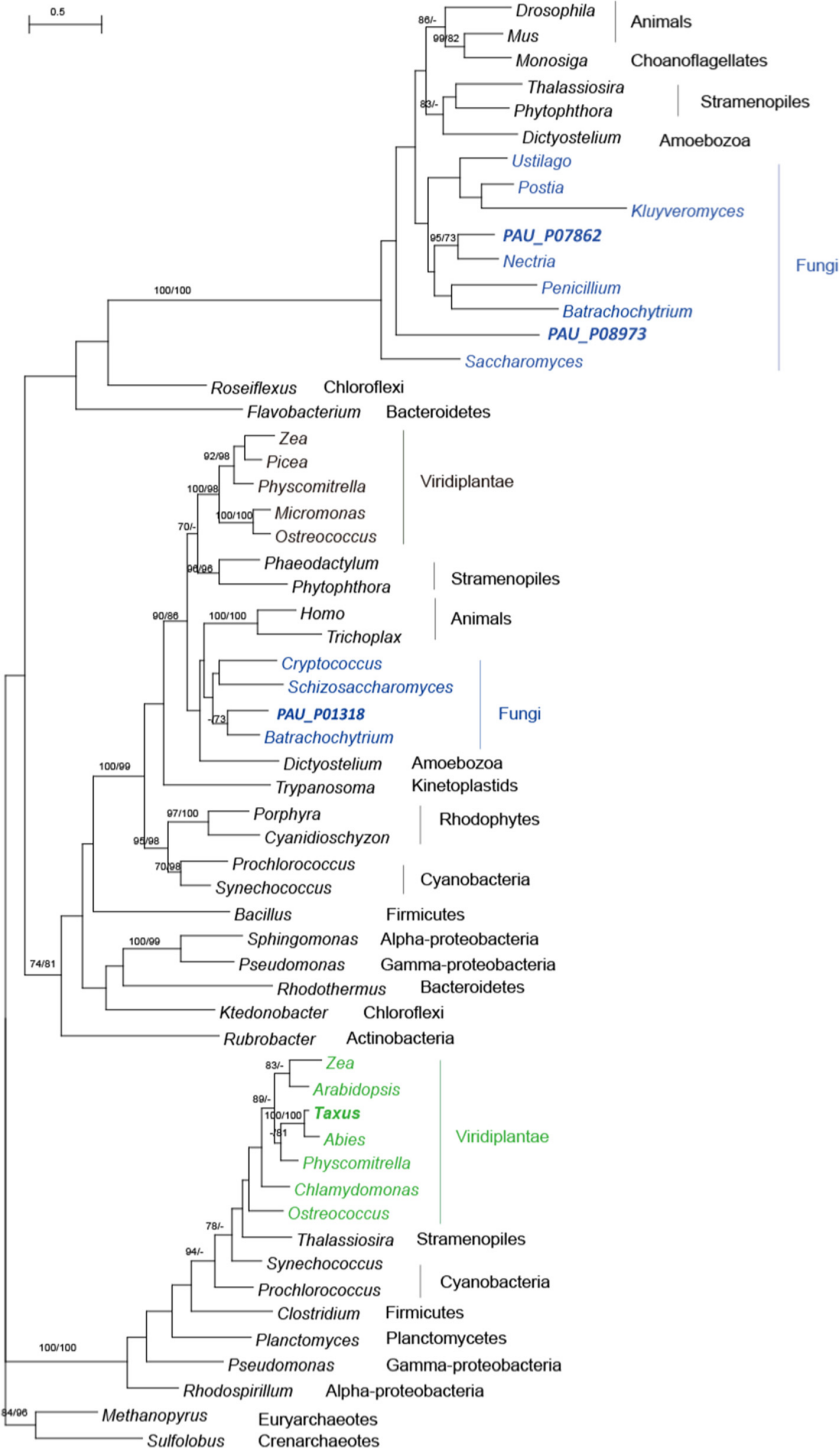
**Molecular phylogeny of GGPPS proteins.** Numbers above branches indicate bootstrap values from maximum likelihood and distance analyses, respectively. Dashes indicate bootstrap values lower than 70%. The GGPPS of *Taxus* and the homologs in *P. aurantiogriseum* NRRL 62431 were in black font. The taxa belonging to Viridiplantae and Fungi were shown in green and blue.

The *GGPPS* in green plants formed a strong clade with those from cyanobacteria, which implies the endosymbiotic gene transfer likely took place in the common ancestor of green plants. PAU_P07862, PAU_P08973 in *P. aurantiogriseum* NRRL 62431 and the biochemically characterized *GGPPS* in fungi *P. paxilli* clustered with the potential homologs from animals, choanoflagellates, stramenopiles, and some bacteria, which suggested a bacterial origin as the common ancestor of these eukaryotes. Another gene PAU_P01318 in *P. aurantiogriseum* NRRL 62431, which shows 35% identity with *Taxus GGPPS*, was also included in our phylogenetic analysis. This gene and other similar eukaryotic genes formed a strongly supported clade, suggesting a distinctly different origin from the above *GGPPSs* (Figure 3).

**Figure 4:**
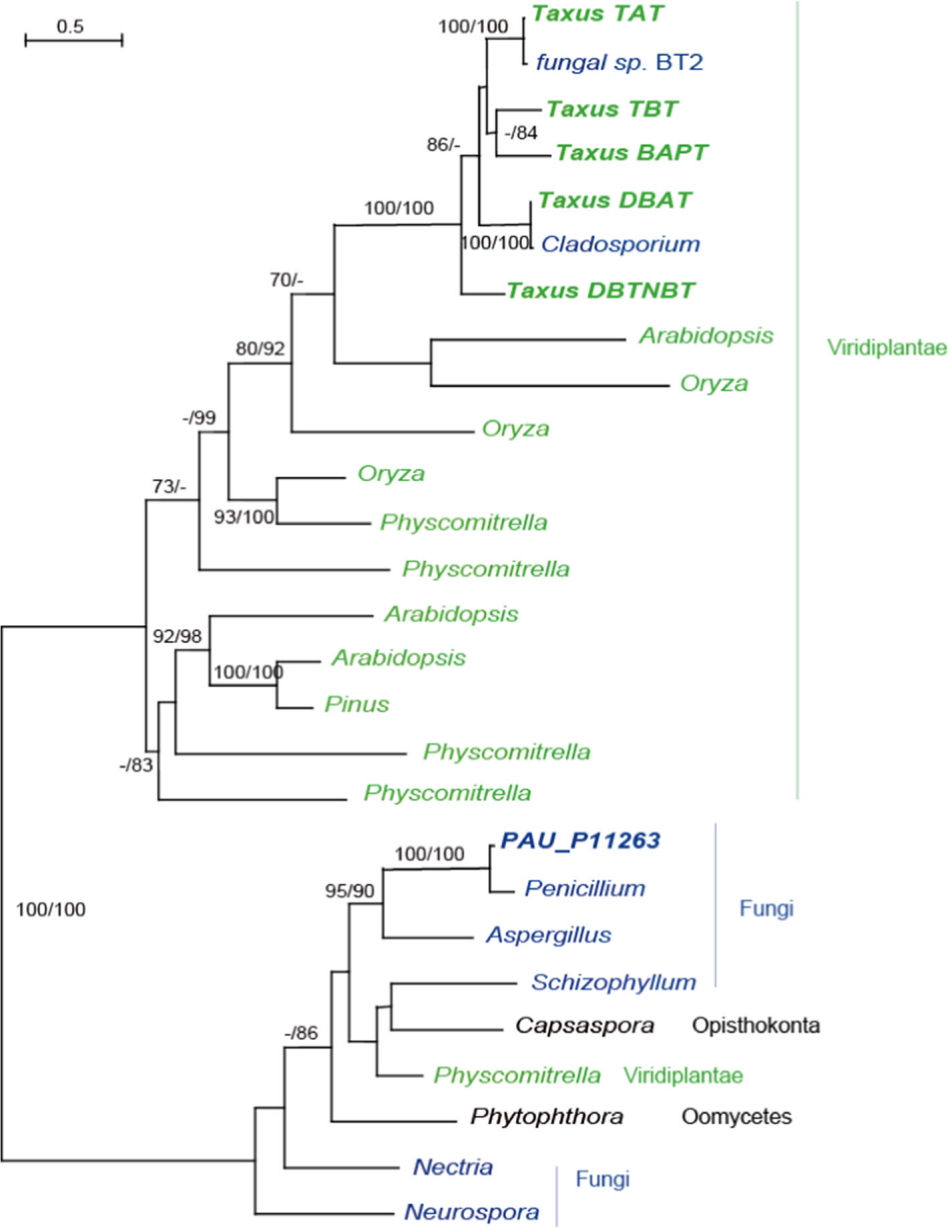
**Molecular phylogeny of acyltransferase proteins.** Numbers above branches indicate bootstrap values from maximum likelihood and distance analyses, respectively. Dashes indicate bootstrap values lower than 70%. The sequence in *Taxus* was in black font. The taxa belonging to Viridiplantae and Fungi were shown in green and blue, respectively.

**Figure 5:**
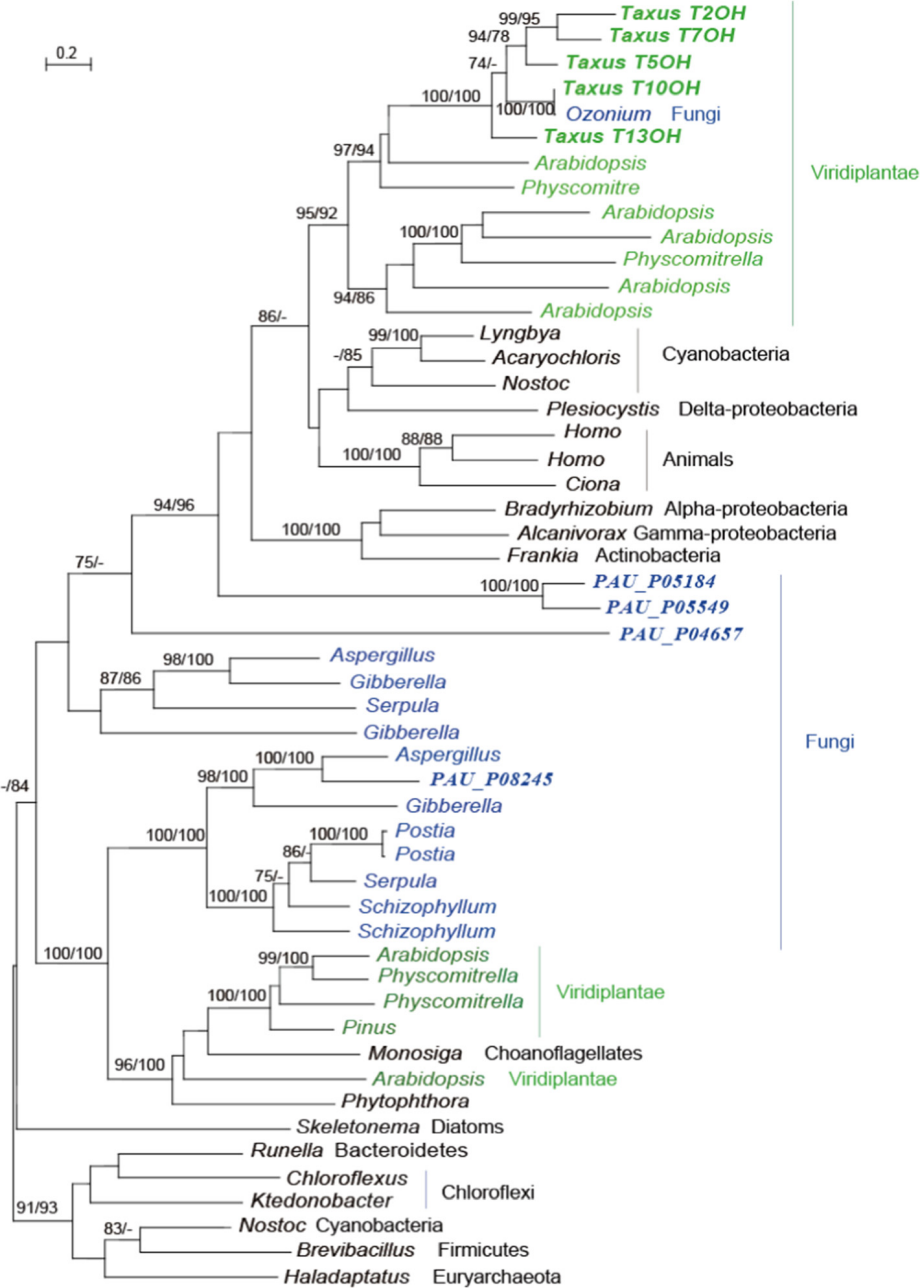
**Molecular phylogeny of hydroxylase proteins.** Numbers above branches indicate bootstrap values from maximum likelihood and distance analyses, respectively. Dashes indicate bootstrap values lower than 70%. The sequence in *Taxus* was in black font. The taxa belonging to Viridiplantae and Fungi were shown in green and blue, respectively.

Genes with high similarity to acyltransferases and P450s in green plants and fungi, including *P. aurantiogriseum* NRRL 62431, formed distinct branches in their own phylogenetic trees (Figures 4 and 5). This suggested their independent evolution in plants and fungi. All the acyltransferases and P450 in *Taxus* clustered together, suggesting that recent gene duplication took place after the split of *Taxus* from other plants.

**Figure 6:**
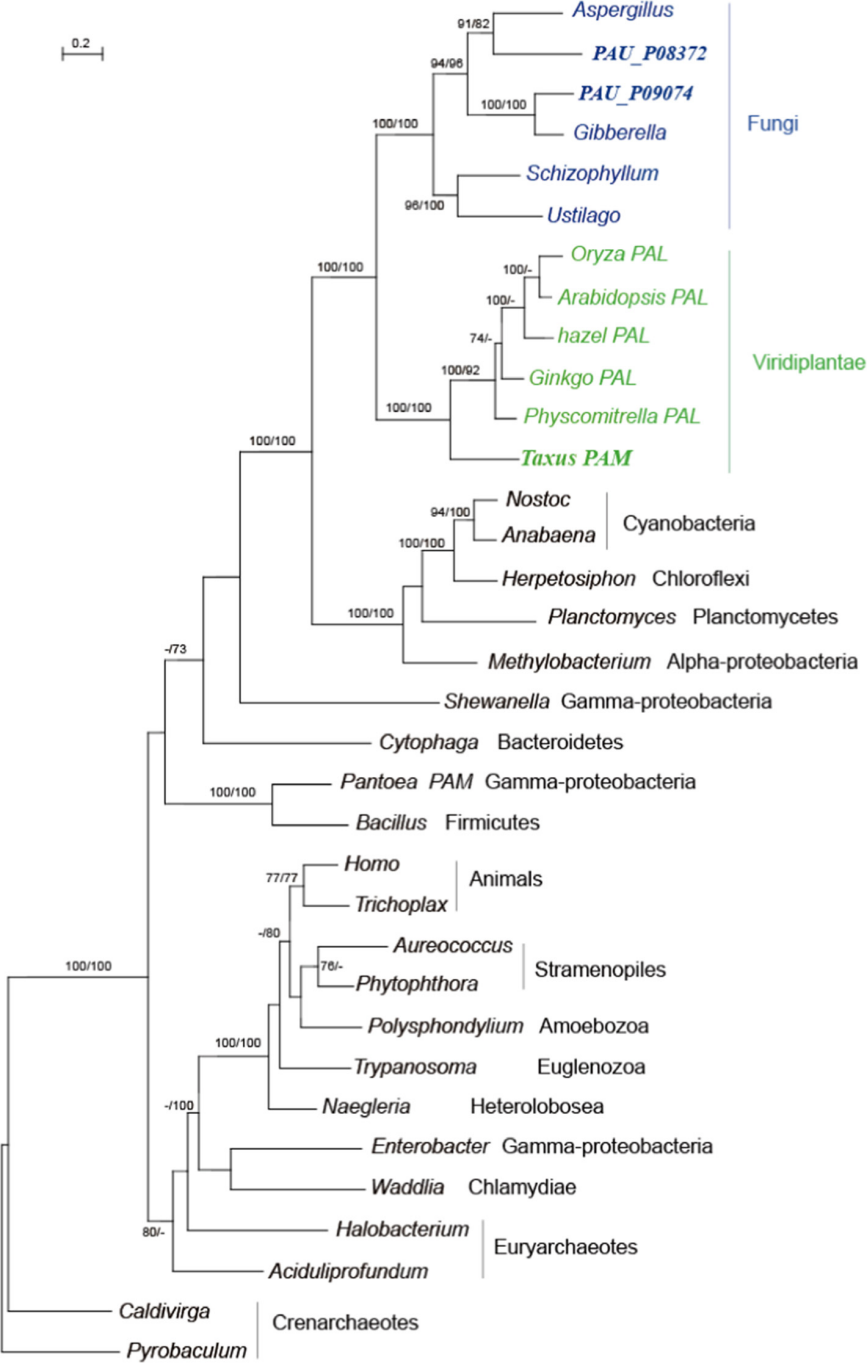
**Molecular phylogeny of PAM proteins.** Note that the genes in plants other than *Taxus* are annotated as phenylalanine ammonia-lyase (PAL) in GenBank. Numbers above branches indicate bootstrap values from maximum likelihood and distance analyses, respectively. Dashes indicate bootstrap values lower than 70%. The sequence in *Taxus* was in black font. The taxa belonging to Viridiplantae and Fungi were shown in green and blue, respectively.

The phylogenetic tree constructed reveals that *Taxus PAM* cluster as a sister branch of *PAL* (phenylalanine ammonia-lyase) in land plants and further formed a clade with homologs from fungi including *P. aurantiogriseum* NRRL 62431 (Figure 6). The homologs from animals and other eukaryotes showed a highly supported clade within bacterial taxa (Figure 6), suggesting a different prokaryotic origin from that in plants and fungi. Given the wide prevalence of PAM and its possible function in other pathways, ancient gene transfer between plants and fungi may have happened in the ancestors of the plants and fungi, with the transfer direction and other details unknown.

## Discussion

Biosynthesis of paclitaxel in *Taxus* is thought to involve 19 steps from geranylgeranyl diphosphate and 13 genes involved in paclitaxel biosynthesis have been identified and well characterized. However, little is known about the taxol biosynthetic genes in the endophytic fungi or their evolutionary origin. Recently it was controversially suggested that paclitaxel synthesis detected in a range of fungal endophytes was a result of residual taxanes synthesized by the host [15]. However this theory ignores the discovery of paclitaxel synthesizing endophytic fungi found on non-paclitaxel hosts [28] and that Stierle et al.in their seminal work demonstrated de novo synthesis of paclitaxel occurred in pure fungal endophyte cultures using both [1-^14^C] acetic acid and L-[U-^14^C] phenylalanine as precursors [11]. We found relatively small amounts of paclitaxel was normally synthesized by *P. aurantiogriseum* NRRL 62431, but that the level was increased about 5-fold, from 0.07 mg/L to 0.35 mg/L with the addition of methyl jasmonate and phenylalanine to the culture medium. In addition, the fungal cells used in our study did not have contact with the host plant for more than twelve passages, again refuting the possibility that paclitaxel from *P. aurantiogriseum* NRRL 62431 occurred via passive release of taxanes accumulated in endophytic fungal cell walls from its host hazel.

In order to provide insight into the evolutionary origins of paclitaxel synthesis we sequenced the genome of *P. aurantiogriseum* NRRL 62431. Potential gene candidates involved in paclitaxel biosynthesis were identified by homology with existing paclitaxel biosynthetic genes from *Taxus*. The independent origin of *GGPPS*, acyltransferase, P450 and *PAM* in the endophytic *P. aurantiogriseum* NRRL 62431 and in the host *Taxus* were universally supported by the distinct conserved amino acid sites in the multiple sequence alignments (Additional file 1: Figures S3, S4, S5 and S6). This data supports the findings of Xiong et al., who found only a 40.6% identity of nucleic acid sequence between *T. media* and the putative *TS* from endophytic fungi isolated from *T. media*, and 44.1% identity between putative *BAPT* segments and the *T. media* gene [17]. The high similarities of the previously identified sequences of *TS*, *BAPT* in *T. andreanae*[29] and *DBAT* in *Cladosporium cladosporioides* MD2 [12] with the homologs in *Taxus* (more than 97%) that fueled speculation about the origins of paclitaxel biosynthesis in fungi, are likely to represent potential cross contamination between endophytic fungi and host DNA.

There is precedent for the independent development of the same biosynthetic pathway in plants and fungi. Like paclitaxel, gibberellins (GAs) are complex diterpenoid compounds. GAs were first isolated as metabolites from rice fungal pathogen *Gibberella fujikuroi* (now is renamed as *Fusarium fujikuroi*) [30]. Although *F. fujikuroi* and higher plants produce structurally identical GAs, profound differences have been found in the GA pathways and enzymes of plants and fungi [31]. The substantial differences in genes and enzymes indicate that plants and fungi have evolved their complex GA biosynthesis pathways independently and the possibility of horizontal gene transfer of GA genes between the plants and the fungi is highly unlikely [32]. A similar situation seems to have taken place in the paclitaxel biosynthetic pathway in fungi and plants. Only 7 potential homologs to the 13 known paclitaxel biosynthetic genes were identified from *P. aurantiogriseum* NRRL 62431 or *F. solani,* supporting the divergence of the two biosynthetic pathways. The fact that putative candidates for some of the steps in paclitaxel synthesis can be found in fungi with the ability to synthesize paclitaxel suggests that only specific enzymatic sites associated with enzymatic activity might be conserved, while the overall protein structure may differ.

In the past few years, some efforts have been made worldwide to engineer fungi by transferring paclitaxel biosynthetic genes in *Taxus* to fungi. Most metabolic engineering attempts were based on the assumption that paclitaxel biosynthetic genes in fungus and *Taxus* plant are interchangeable [33]. However, such metabolic engineering attempts have not been successful. Although candidate genes involved in paclitaxel biosynthesis still need be biochemically characterized, evidence from our genome study provides a greater understanding of their evolutionary origins. This understanding may result in a better-informed engineering approach that significantly improves paclitaxel biosynthesis.

## Conclusions

Our results demonstrate that paclitaxel biosynthetic gene candidates in endophytic fungus *P. aurantiogriseum* NRRL 62431 are quite different from those in hosts *C. avellana* and *T. baccata* in terms of amino acid sequences and may have a distinctly different evolutionary pattern. The relationship between paclitaxel biosynthetic genes in *P. aurantiogriseum* NRRL 62431 and the homologs in its hosts are more complex than expected, and we have provided evidence that horizontal gene transfer is unlikely to have occurred. The genomic resources generated in our study provide new insights into the evolution of enzymes that might involve in the biosynthesis of paclitaxel in fungi and will likely facilitate production of larger quantities of this compound from fungi for the treatment of cancer patients.

## Methods

### Strain and culture conditions

Fungal cultures were isolated from freshly harvested *Corylus avellana* “Barcelona” nuts from Aurora, Oregon, USA. The fungal isolate was identified as *Penicillium aurantiogriseum* by Dr. Frank Dugan, Research Plant Pathologist, USDA-ARS Western Regional Plant Introduction Station and deposited in the NRRL database as NRRL 62431. A 1 week-old sporulating culture on PDA was rinsed with 20 mL of sterile water containing 1 drop of Tween-20. Two mL of the spore solution with an absorbance of about 0.8 at 600 nm was added to each of 6 liters of potato dextrose broth (PDB, DIFCO). Broth cultures were shaken at 20°C and 100 rpm for 2 weeks. On Day 14, when the amount of reducing sugars in the cultures was no longer detectable using glucose test strips, 100 μL of methyl jasmonate and 0.172 g/L filter-sterilized phenylalanine were added to each flask, and shaking was resumed. The cultures were harvested on day 24.

### Taxane identification and purification

Mycelia were filtered from broth using vacuum filtration. Culture broth was extracted with dichloromethane and mycelia was freeze-dried, pulverized and extracted with dichloromethane. Solvent was removed by reduced pressure at 36°C and the extracts were pooled. To the final crude extract, 0.344 g, 50 mL of water was added, and the mixture separated on C-18 cartridge (Fisher) with vacuum. The methanol solution was dried (0.256 g) and dissolved in methanol or acetonitrile to 200 μg/μL after which it was filtered through a 0.45 nm filter. Analyses were performed with a Shimadzu 2010 HPLC-MS (APCI or ESI) system and a diode array detector. The sample was fractionated and collected several times by HPLC on a Phenomenex Curosil PFP column (250 mm × 4.6 mm) at 40°C. Mobile phases were (A) 10 mM ammonium acetate, pH 4.0 and (B) HPLC- grade acetonitrile (J.T. Baker). The flow rate was isocratic at 1 mL/min (or 2.5 ml/min for the preparative column), 50% of each eluent. The UV detector was set at 254 and 228 nm. The crude sample was fractionated, and mass signatures of baccatin III, cephalomannine and paclitaxel were detected. Calibration curves were made for these three taxanes using authentic standards, and the approximate amount of each recovered per liter of culture was calculated. Fractions were collected from the entire extract at the times expected for taxanes determined with MS at approximately 7, 13 and 15 minutes ± 1 minute. About 120 μg of purified paclitaxel was recovered. Mass spectrum and ^1^H NMR (Varian 400 MHz) were used to confirm the presence of paclitaxel. In addition to paclitaxel, cephalomannine and baccatin III were identified from their mass spectra and retention times of authentic standards. EI-MS for cephalomannine: *m/z* (% rel. int.) 832 (75) [M+]. 754 (25). 569 (65), 551 (50), 509 (95). 264 (100); EI-MS for baccatin III: m/z (% rel. int.) 587 (56) [M+], 527 (100), 509 (50), 405 (44), 327.

### Genome sequencing, assembly and annotation

Mycellium of *P. aurantiogriseum* strain (NRRL 62431) were harvested and immediately frozen in liquid nitrogen. The materials were stored in a −80°C freezer until DNA extraction. Genomic DNA for construction of libraries was isolated from fungal using the CTAB method reported by Goodwin et al. [34]. Libraries were constructed following the standard Illumina protocol (Illumina, San Diego, CA, USA). In brief, 5 μg of genomic DNA was fragmented to less than 800 bp using a nebulization technique. The ends of DNA fragments were then repaired by T4 DNA polymerase and the *E. coli* DNA polymerase I Klenow fragment added an overhang “A” bases. DNA fragments were ligated to PCR and sequencing adaptors, and then were purified in 2% agarose gels to separate and collect ~400 bp fragments. The resulting DNA templates were enriched by 18 cycles of PCR. The libraries were sequenced on an Illumina GA2 generating 59,951,610 reads of 100 bases in length. The generated reads were inspected and poor quality reads/bases were removed. High quality reads were then assembled using ABySS 1.2.1. with various k-mer sizes ranging from 50 to 63. The optimal k-mer size was empirically set to 54 and the resulting assembled sequences were used for downstream analyses. Gene models were predicted using GeneMark [21], TWINSCAN [22] and GeneWise [23]. Contigs with at least 1000 bp were searched against nr protein database using BLASTx. Genomic sequences with 90% identity that spanned more than 80% of a protein were extended 500 bp up and downstream and passed to GeneWise to predict gene models. A total of 2901 gene models were obtained and termed ‘GW gene models’. An *ab initio* prediction was conducted using a combination of GeneMark and TWINSCAN. Two set of gene models were predicted using GeneMark and TWINSCAN separately yielding 11,793 and 10,981 gene models, respectively. These datasets were then merged to build a reference gene model set. Gene models were then clustered to generate ‘gene clusters’. Next, a representative gene model sequence for each gene cluster was selected based on best E value matches, sequence identity and coverage of nr proteins (non-redundant proteins of NCBI). These representative gene models will be called ‘AB gene models’. GW gene models and AB gene models were then combined to build the final gene model dataset composed of 11,476 gene models. Putative functions of gene models were predicted by aligning proteins to the NCBI nr database using blast2GO [35]. tRNAs were predicted using tRNAscan-SE [36]. Putative protein domains and GO analysis were assigned using Agbase [37]. Transposons and repeat sequences were determined using RepeatMasker [38].

### Transcriptome sequencing

The mature seeds of hazel (*C. avellana* L.) and *Taxus chinensis* shoots were collected. Total RNA was isolated according to the method described by Chang et al. [39]. The mRNA was purified from 10 μg of total RNA by using oligo (dT) _25_ magnetic beads. After purification from total RNA, the resulting mRNA was fragmented into small pieces. The RNA fragments were used for first strand cDNA synthesis using random primers. Second strand cDNA synthesis was conducted by adding DNA polymerase I and RNase H. The cDNA products were purified with a QIAquick PCR Purification Kit. The purified cDNA fragments went through an end repair process and were then ligated to polyA and the adapters. The ligation products were purified with a QIAquick Gel Extraction Kit and were further enriched with PCR for creating the final cDNA library. The library was separated on gel and fragments between 250-300 bp were harvested and purified with a QIAquick Gel Extraction Kit. Sequencing was performed on the Illumina genome Analyzer. Image deconvolution and quality value calculations were conducted using the Illumina GA pipeline 1.3. Empty reads, adaptor sequences and low quality sequences (reads with ambiguous bases ‘N’) were removed and then high quality reads were further randomly clipped into 21 bp K-mers for de novo assembly. SOAPdenovo was used to assemble the transcriptome sequences [40]. Distinct unigene sequences were used for blast search and annotation against NCBI nr, NCBI nt, COG, KEGG and Swiss-Prot database with an E-value cut-off of 1e-05. Functional annotation of GO terms (http://www.geneontology.org) was performed by Blast2GO software [35]. Unigenes’ GO functional classification was performed using WEGO tool [41]. Pathway annotations were analyzed using Blastall. Annotation of peptide sequence was done by searching transcripts against the NCBI non-redundant (nr) peptide database which includes all non-redundant GenBank CDS translations, RefSeq Proteins, PDB, Swiss-Prot, PIR and PRF. The search was conducted using BLASTx with an E-value cut-off of 1e-05 and matching to the top hits. Prediction of CDS was also done using the ESTScan software [42].

### Comparative analyses between *Penicillium aurantiogriseum* NRRL 62431 with hazel (*C. avellana* L.), *Taxus baccata* and *EF0021*

Paclitaxel biosynthetic genes in hazel were compared against *P. aurantiogriseum* NRRL 62431 proteins using native BLASTx with an E-value cut-off of 1e-05.

The 454 sequencing data of transcriptome of *T. baccata* was retrieved from NCBI SRA database (http://trace.ncbi.nlm.nih.gov/Traces/sra, SRA number SRX026383). The SRA file was converted to fasta format using SRA toolkits (http://trace.ncbi.nlm.nih.gov/Traces/sra/std). 454 reads were assembled using Newbler (version 2.5) with default parameters. Contigs with length >100 bp were used for further analysis. The functional annotation of the transcriptome of *T. baccata* was conducted using BLAST2GO [35]. Paclitaxel biosynthetic genes in *T. baccata* were compared against *P. aurantiogriseum* NRRL 62431 proteins using native BLASTx with an E-value cut-off of 1e-05.

The DNA contigs sequences from the EF0021 genome sequence were retrieved from GenBank (PRJNA77807). Putative paclitaxel biosynthetic genes from *P. aurantiogriseum* NRRL 62431 were compared against the EF0021 DNA contigs using native tBLASTn with an E-value cut-off of 1e-05.

### Phylogenetic analysis

The amino acid sequence of the 13 reported enzymes involved in paclitaxel biosynthesis in *Taxus* spp. (Additional file 2: Table S1) were used as queries to search against all the putative proteins in *P. aurantiogriseum*, and then all the hits were used as queries to search against GenBank nr database and the proteins were kept for the following phylogenetic analyses if their hits were annotated as the same proteins or belong to the same protein families. Besides the sequences obtained above, homologs used for phylogenetic tree construction were retrieved from GenBank nr database. To get a comprehensive view about the gene evolution, we performed multiple separate blast searches by restricting the database to the sequences from fungi, animals, and other eukaryotic groups or by excluding the sequences from land plants and/or fungi, and the sequences from representative species were added to the previous dataset. In some case, PSI-BLAST was used to obtain the homologs with low similarities. Protein sequence alignment was performed using ClustalX, followed by manual refinement. Alignments are deposited in TreeBase (ID S15183) [43].Truncated sequences and those sequences with poor identities were removed, gaps and ambiguously sites in the alignment were weeded by visual inspection. The protein substitution matrix, rate heterogeneity and invariable sites were rated using ModelGenerator [44] for each protein and the most appropriate model was chosen. Phylogenetic analyses were carried out with a maximum likelihood method using PHYML [45] and a distance method using neighbor of PHYLIPNEW v.3.68 in EMBOSS package [45,46]. For distance analyses, maximum likelihood distances were calculated using TREE-PUZZLE v.5.2 [47] and PUZZLEBOOT v.1.03 (A. Roger and M. Holder, http://www.tree-puzzle.de). Because the LG model has not been applied in TREE-PUZZLE, the next available model was used in distance calculation. Bootstrap support values for both methods were estimated using 100 pseudo-replicates.

## Availability of supporting data

Raw sequencing data, assembly and annotations of *P. aurantiogriseum* NRRL 62431 genome have been deposited in GenBank under the accession [GenBank: SRA056290 and ALJY00000000]. Raw transcriptome sequencing data, assembly and annotations of *C. avellana* have been deposited in GenBank under the accessions [GenBank: SRA056312 and KA393721-KA430773] respectively.

Phylogenic alignments have been deposited in TreeBase; submission ID S15183, (http://purl.org/phylo/treebase/phylows/study/TB2:S15183?x-access-code=5451b71164255d696111850e35d82559&format=html).

## Abbreviations

BAPT: Baccatin III: 3-animo-3-phenylpropanoyltransferase; DBAT: 10-deacetylbaccatin III-10-O-acetyltransferase; DBTNBT: 3′-N-debenzoyl-2′-deoxytaxol N-benzoyltransferase; GGPPS: Geranylgeranyl diphosphate synthase; PAM: Phenylalanine aminomutase; TAT: Taxadien-5α-ol-O-acetyl transferase; TBT: Taxane 2α-O-benzoyltransferase; TS: Taxa-4(5),11(12)-diene synthase; T1OH: Taxane 1β-hydroxylase; T2OH: Taxane 2α-hydroxylase; T5OH: Cytochrome P450 taxadiene 5α-hydroxylase; T7OH: Taxane 7β-hydroxylase; T10OH: Taxane 10β-hydroxylase; T13OH: Taxane 13α-hydroxylase.

## Competing interests

The authors declare that they have no competing interests.

## Author contributions

DQ, JL, AH, KDW designed the research of the project. QT, XH prepared the genomic DNA of *P. aurantiogriseum*. QT, HL, LC collected plant material and isolated RNA samples for transcriptome sequencing. YY, HZ, GS, RAB, BZ, GG and YZ carried out bioinformatics analysis, AH, JWP, RB isolated fungus and analyzed taxanes. JL and FX assisted the bioinformatics analysis. DQ, IW, RAB, MIB and KDW conducted data analysis and wrote the manuscript. All authors read and approved the final manuscript.

## Supplementary Material

Additional file 1 **Figure S1.** Overview of paclitaxel biosynthetic pathway in *Taxus* sp. Abbreviations: BAPT: baccatin III: 3-animo-3-phenylpropanoyltransferase; DBAT: 10-deacetylbaccatin III-10-O-acetyltransferase; DBTNBT: 3′-N-debenzoyl-2′-deoxytaxol N-benzoyltransferase; GGPPS: geranylgeranyl diphosphate synthase; PAM: phenylalanine aminomutase; TAT: taxadien-5α-ol-O-acetyl transferase; TBT: taxane 2α-O-benzoyltransferase; TS: taxa-4(5),11(12)-diene synthase; T1OH: Taxane 1β-hydroxylase; T2OH: taxane 2α-hydroxylase; T5OH: cytochrome P450 taxadiene 5α-hydroxylase;T7OH: taxane 7β-hydroxylase; T10OH:taxane 10β-hydroxylase; T13OH: taxane 13α-hydroxylase. Genes responsible for C1 hydroxylation, oxetane formation, C9 oxidation, β-phenylalanoyl CoA formation and C2′hydroxylation of paclitaxel biosynthetic pathway in *Taxus* sp. remains to be elucidated (marked with red colour). **Figure S2** HPLC-MS analysis of paclitaxel from the extracts of *P. aurantiogriseum* culture. (a) Total ion current (TIC) for the purified fungal extract. HPLC chromatogram showing the retention times and relative peak areas of the taxanes extracted from the *P. aurantiogriseum* culture medium. (b) Mass spectrum of paclitaxel extracted from total ion current. **Figure S3** Sequence alignments of GGPPS in the endophytic *P. aurantiogriseum* NRRL 62431 and in the host *Taxus*. **Figure S4** Sequence alignments of acyltransferases in the endophytic *P. aurantiogriseum* NRRL 62431 and in the host *Taxus*. **Figure S5** Sequence alignments of P450 in the endophytic *P. aurantiogriseum* NRRL 62431 and in the host *Taxus*. **Figure S6** Sequence alignments of PAM in the endophytic *P. aurantiogriseum* NRRL 62431 and in the host *Taxus*. Click here for file

Additional file 2 **Table S1.** List of the 13 reported paclitaxel biosynthetic enzymes in *Taxus* sp. **Table S2** Gene ontology classification of *P. aurantiogriseum* NRRL 62431 genes. Click here for file

Additional file 3 **Data S1.** Transcription factors in the genome of *Penicillium aurantiogriseum* NRRL 62431. **Data S2** Transporters in the genome of *Penicillium aurantiogriseum* NRRL 62431. **Data S3** Blast result of the 13 reported paclitaxel biosynthetic genes in *Taxus sp*. against the genome of *Penicillium aurantiogriseum* NRRL 62431. **Data S4** Blast result of the 13 reported paclitaxel biosynthetic genes in *Taxus sp*. against the unigenes in the transcriptome of Hazel (*Corylus avellana* L.). **Data S5** DNA sequences of paclitaxel biosynthetic gene candidates in Hazel (*Corylus avellana L*.). **Data S6** Blast results of Hazel paclitaxel biosynthetic gene candidates against *Penicillium aurantiogriseum* NRRL 62431 proteins. **Data S7** DNA sequences of paclitaxel biosynthetic genes in *Taxus baccata*. **Data S8** Blast result of paclitaxel biosynthetic genes of *Taxus baccata* against *Penicillium aurantiogriseum* NRRL 62431 proteins. **Data S9** Blast result of the paclitaxel biosynthetic genes candidates in *Penicillium aurantiogriseum* NRRL 62431 against the genome of EF0021. Click here for file
