# Supplementary material for: Genome sequencing and analysis of the paclitaxel-producing endophytic fungus Penicillium aurantiogriseum NRRL 62431
Source: BMC Genomics. 2014 Jan 25;15:69. doi: 10.1186/1471-2164-15-69 (PMC3925984; doi:10.1186/1471-2164-15-69)
Supplement: Additional file 1 — Figure S1. Overview of paclitaxel biosynthetic pathway in Taxus sp. Abbreviations: BAPT: baccatin III: 3-animo-3-phenylpropanoyltransferase; DBAT: 10-deacetylbaccatin III-10-O-acetyltransferase; DBTNBT: 3′-N-debenzoyl-2′-deoxytaxol N-benzoyltransferase; GGPPS: geranylgeranyl diphosphate synthase; PAM: phenylalanine aminomutase; TAT: taxadien-5α-ol-O-acetyl transferase; TBT: taxane 2α-O-benzoyltransferase; TS: taxa-4(5),11(12)-diene synthase; T1OH: Taxane 1β-hydroxylase; T2OH: taxane 2α-hydroxylase; T5OH: cytochrome P450 taxadiene 5α-hydroxylase;T7OH: taxane 7β-hydroxylase; T10OH:taxane 10β-hydroxylase; T13OH: taxane 13α-hydroxylase. Genes responsible for C1 hydroxylation, oxetane formation, C9 oxidation, β-phenylalanoyl CoA formation and C2′hydroxylation of paclitaxel biosynthetic pathway in Taxus sp. remains to be elucidated (marked with red colour). Figure S2 HPLC-MS analysis of paclitaxel from the extracts of P. aurantiogriseum culture. (a) Total ion current (TIC) for the purified fungal extract. HPLC chromatogram showing the retention times and relative peak areas of the taxanes extracted from the P. aurantiogriseum culture medium. (b) Mass spectrum of paclitaxel extracted from total ion current. Figure S3 Sequence alignments of GGPPS in the endophytic P. aurantiogriseum NRRL 62431 and in the host Taxus. Figure S4 Sequence alignments of acyltransferases in the endophytic P. aurantiogriseum NRRL 62431 and in the host Taxus. Figure S5 Sequence alignments of P450 in the endophytic P. aurantiogriseum NRRL 62431 and in the host Taxus. Figure S6 Sequence alignments of PAM in the endophytic P. aurantiogriseum NRRL 62431 and in the host Taxus. [file 1471-2164-15-69-S1.zip › 2078393261107576_add3.doc]

**Figure S1. Overview of paclitaxel biosynthetic pathway in *Taxus* sp**. Abbreviations: BAPT: baccatin III: 3-animo-3-phenylpropanoyltransferase; DBAT: 10-deacetylbaccatin III-10-O-acetyltransferase; DBTNBT: 3′-N-debenzoyl-2′-deoxytaxol N-benzoyltransferase; GGPPS: geranylgeranyl diphosphate synthase; PAM: phenylalanine aminomutase; TAT: taxadien-5α-ol-O-acetyl transferase; TBT: taxane 2α-O-benzoyltransferase; TS: taxa-4(5),11(12)-diene synthase; T1OH: Taxane 1β-hydroxylase; T2OH: taxane 2α-hydroxylase; T5OH: cytochrome P450 taxadiene 5α-hydroxylase;T7OH: taxane 7β-hydroxylase; T10OH:taxane 10β-hydroxylase; T13OH: taxane 13α-hydroxylase. Genes responsible for C1 hydroxylation, oxetane formation, C9 oxidation, β-phenylalanoyl CoA formation and C2`hydroxylation of paclitaxel biosynthetic pathway in *Taxus* sp. remains to be elucidated (marked with red colour).
