# Supplementary material for: Genome sequencing and analysis of the paclitaxel-producing endophytic fungus Penicillium aurantiogriseum NRRL 62431
Source: BMC Genomics. 2014 Jan 25;15:69. doi: 10.1186/1471-2164-15-69 (PMC3925984; doi:10.1186/1471-2164-15-69)
Supplement: Additional file 2 — Table S1. List of the 13 reported paclitaxel biosynthetic enzymes in Taxus sp. Table S2 Gene ontology classification of P. aurantiogriseum NRRL 62431 genes. [file 1471-2164-15-69-S2.zip › 2078393261107576_add1.doc]

**Table S1. List of the 13 reported paclitaxel biosynthetic enzymes in *Taxus* sp.**

| **Enzyme** | **Abbreviations** | **Accession No.** | **CDS**  **(bp)** |
| --- | --- | --- | --- |
| Baccatin III: 3-animo-3-phenylpropanoyltransferase | BAPT | AY082804 | 1,335 |
| 10-deacetylbaccatin III-10-O-acetyltransferase | DBAT | AF193765 | 1,320 |
| 3′-N-debenzoyl-2′-deoxytaxol N-benzoyltransferase | DBTNBT | AF466397 | 1,323 |
| Geranylgeranyl diphosphate synthase | GGPPS | AF081514 | 1,182 |
| Phenylalanine aminomutase | PAM | AY582743 | 2,094 |
| Taxane 10-beta hydroxylase | T10OH | AF318211 | 1,494 |
| Taxane 13-alpha hydroxylase | T13OH | AY056019 | 1,458 |
| Taxane 2-alpha hydroxylase | T2OH | AY518383 | 1,488 |
| Taxane 5-alpha hydroxylase | T5OH | AY307951 | 1,503 |
| Taxane 7-beta hydroxylase | T7OH | AY289209 | 1,509 |
| Taxadien-5α-ol-O-acetyl transferase | TAT | AF190130 | 1,317 |
| Taxane 2α-O-benzoyltransferase | TBT | AF297618 | 1,320 |
| Taxadiene synthase | TS | AY364469 | 2,586 |
| Taxadiene synthase | TS | U48796 | 2,586 |
