# Supplementary material for: Genome sequencing and analysis of the paclitaxel-producing endophytic fungus Penicillium aurantiogriseum NRRL 62431
Source: BMC Genomics. 2014 Jan 25;15:69. doi: 10.1186/1471-2164-15-69 (PMC3925984; doi:10.1186/1471-2164-15-69)
Supplement: Additional file 2 — Table S1. List of the 13 reported paclitaxel biosynthetic enzymes in Taxus sp. Table S2 Gene ontology classification of P. aurantiogriseum NRRL 62431 genes. [file 1471-2164-15-69-S2.zip › 2078393261107576_add2.doc]

**Table S2**. **Gene ontology classification of *P. aurantiogriseum* NRRL 62431 genes***.*

**A. Biological processs**

| **GO ID** | **GO description** | **# of ocurrences** |
| --- | --- | --- |
| GO:0008152 | metabolic process | 6,296 |
| GO:0008150 | Biological process | 5,042 |
| GO:0006810 | transport | 1,800 |
| GO:0009058 | biosynthetic process | 1,654 |
| GO:0006139 | nucleobase-containing compound metabolic process | 1,537 |
| GO:0006351 | transcription, DNA-dependent | 1,454 |
| GO:0050789 | regulation of biological process | 920 |
| GO:0044238 | primary metabolic process | 838 |
| GO:0005975 | carbohydrate metabolic process | 595 |
| GO:0009056 | catabolic process | 576 |
| GO:0006520 | cellular amino acid metabolic process | 415 |
| GO:0006464 | protein modification process | 380 |
| GO:0019538 | protein metabolic process | 371 |
| GO:0006412 | translation | 346 |
| GO:0006259 | DNA metabolic process | 291 |
| GO:0006629 | lipid metabolic process | 270 |
| GO:0007165 | signal transduction | 237 |
| GO:0006950 | response to stress | 233 |
| GO:0006811 | ion transport | 221 |
| GO:0015031 | protein transport | 170 |
| GO:0016043 | cellular component organization | 100 |
| GO:0006996 | organelle organization | 92 |
| GO:0007049 | cell cycle | 73 |
| GO:0006091 | generation of precursor metabolites and energy | 70 |
| GO:0008219 | cell death | 32 |
| GO:0007010 | cytoskeleton organization | 27 |
| GO:0019725 | cellular homeostasis | 22 |
| GO:0007154 | cell communication | 21 |
| GO:0007005 | mitochondrion organization | 13 |
| GO:0040029 | regulation of gene expression, epigenetic | 12 |
| GO:0009653 | anatomical structure morphogenesis | 9 |
| GO:0009628 | response to abiotic stimulus | 8 |
| GO:0019748 | secondary metabolic process | 8 |
| GO:0009607 | response to biotic stimulus | 7 |
| GO:0009605 | response to external stimulus | 6 |
| GO:0030154 | cell differentiation | 6 |
| GO:0000003 | reproduction | 5 |
| GO:0007275 | multicellular organismal development | 2 |
| GO:0008283 | cell proliferation | 2 |
| GO:0016032 | viral reproduction | 1 |
| GO:0044403 | symbiosis, encompassing mutualism through parasitism | 1 |
| **Total** |  | **24,163** |

B. Molecular function

| **GO ID** | **GO description** | **# of ocurrences** |
| --- | --- | --- |
| GO:0003824 | catalytic activity | 4,595 |
| GO:0005488 | binding | 4,451 |
| GO:0016787 | hydrolase activity | 2,669 |
| GO:0016740 | transferase activity | 2,166 |
| GO:0000166 | nucleotide binding | 1,709 |
| GO:0005215 | transporter activity | 968 |
| GO:0003677 | DNA binding | 807 |
| GO:0003700 | sequence-specific DNA binding transcription factor activity | 548 |
| GO:0003676 | nucleic acid binding | 480 |
| GO:0008233 | peptidase activity | 443 |
| GO:0004672 | protein kinase activity | 326 |
| GO:0016301 | kinase activity | 313 |
| GO:0009055 | electron carrier activity | 201 |
| GO:0003723 | RNA binding | 180 |
| GO:0005515 | protein binding | 168 |
| GO:0005198 | structural molecule activity | 141 |
| GO:0004518 | nuclease activity | 134 |
| GO:0004871 | signal transducer activity | 105 |
| GO:0008135 | translation factor activity, nucleic acid binding | 89 |
| GO:0030234 | enzyme regulator activity | 82 |
| GO:0030246 | carbohydrate binding | 78 |
| GO:0004721 | phosphoprotein phosphatase activity | 56 |
| GO:0005509 | calcium ion binding | 54 |
| GO:0016209 | antioxidant activity | 52 |
| GO:0004872 | receptor activity | 50 |
| GO:0003774 | motor activity | 36 |
| GO:0008289 | lipid binding | 29 |
| GO:0003779 | actin binding | 27 |
| GO:0005216 | ion channel activity | 26 |
| GO:0003674 | molecular function | 25 |
| GO:0045735 | nutrient reservoir activity | 6 |
| GO:0008092 | cytoskeletal protein binding | 4 |
| GO:0003682 | chromatin binding | 3 |
| GO:0005102 | receptor binding | 2 |
| GO:0019825 | oxygen binding | 2 |
| GO:0005326 | neurotransmitter transporter activity | 1 |
| **Total** |  | **21,026** |

C. Cellular component

| **GO ID** | **GO description** | **# of ocurrences** |
| --- | --- | --- |
| GO:0005575 | cellular component | 2,820 |
| GO:0005623 | cell | 2,815 |
| GO:0005622 | intracellular | 1,234 |
| GO:0005634 | nucleus | 1,013 |
| GO:0005737 | cytoplasm | 729 |
| GO:0043226 | organelle | 485 |
| GO:0043234 | protein complex | 367 |
| GO:0005739 | mitochondrion | 198 |
| GO:0005840 | ribosome | 161 |
| GO:0005783 | endoplasmic reticulum | 121 |
| GO:0005856 | cytoskeleton | 89 |
| GO:0005576 | extracellular region | 88 |
| GO:0005694 | chromosome | 77 |
| GO:0005794 | Golgi apparatus | 51 |
| GO:0005886 | plasma membrane | 51 |
| GO:0005654 | nucleoplasm | 39 |
| GO:0005730 | nucleolus | 35 |
| GO:0016023 | cytoplasmic membrane-bounded vesicle | 23 |
| GO:0005618 | cell wall | 21 |
| GO:0005773 | vacuole | 21 |
| GO:0005768 | endosome | 18 |
| GO:0005777 | peroxisome | 17 |
| GO:0005829 | cytosol | 17 |
| GO:0009536 | plastid | 17 |
| GO:0005635 | nuclear envelope | 15 |
| GO:0009579 | thylakoid | 12 |
| GO:0000228 | nuclear chromosome | 8 |
| GO:0005815 | microtubule organizing center | 6 |
| GO:0005615 | extracellular space | 5 |
| GO:0030312 | external encapsulating structure | 5 |
| GO:0030313 | cell envelope | 5 |
| GO:0005764 | lysosome | 3 |
| GO:0000229 | cytoplasmic chromosome | 1 |
| **Total** | | **10,567** |
